# Coexistence of Myeloid and Lymphoid Neoplasms: A Single-Center Experience

**DOI:** 10.1155/2019/1486476

**Published:** 2019-11-03

**Authors:** Anthi Bouchla, Thomas Thomopoulos, Sotirios Papageorgiou, Panagiotis Tsirigotis, Efthymia Bazani, Konstantinos Gkirkas, Diamantina Vasilatou, Eirini Glezou, Georgia Stavroulaki, Konstantinos Gkontopoulos, George Dimitriadis, Vasiliki Pappa

**Affiliations:** Second Department of Internal Medicine and Research Unit, Hematology Unit, University General Hospital “Attikon”, 1 Rimini St. Haidari, 12462 Athens, Greece

## Abstract

The coexistence of a myeloid and a lymphoid neoplasm in the same patient is a rare finding. We retrospectively searched the records of the Hematology Division of the Second Department of Internal Medicine and Research Institute at Attikon University General Hospital of Athens from 2003 to 2018. Nine cases have been identified in a total of 244 *BCR-/ABL1-* negative MPN and 25 MDS/MPN patients and 1062 LPD patients referred to our institution between 2003 and 2018. Each case is distinct in the diversity of myeloid and lymphoid entities, the chronological occurrence of the two neoplasms, and the patient clinical course. All of them exhibit myeloproliferative (6 *JAK2 V617F*-positive cases) and lymphoproliferative features, with 1 monoclonal B-cell lymphocytosis (MBL), 3 B-chronic lymphocytic leukemias (B-CLL), 3 B-non-Hodgkin lymphomas (B-NHL), 1 multiple myeloma (MM), and 1 light and heavy deposition disease (LHCDD), while in three cases myelodysplasia is also present. The challenges in identifying and dealing with these rare situations in everyday clinical practice are depicted in this article.

## 1. Introduction

The annual incidence of *BCR-/ABL1*-negative myeloproliferative neoplasms (MPN), namely polycythemia vera (PV), essential thrombocythemia (ET), and primary myelofibrosis (PMF) in the western world, is estimated to be maximum 2.8, 2.3, and 1.5 cases per 100.000 population, respectively [1, 2]. On the other hand, the cumulative incidence of lymphoproliferative disorders (LPD) is difficult to be estimated as they comprise a highly heterogenous group; however, that of a major representative of this group, B-chronic lymphocytic leukemia (B-CLL) seems to be around 5.5 cases per 100.000 population per year [3]. Based on the notion that the concomitance of MPN and LPD in the same patient should be the product of the individual incidence rates, an extremely low incidence of cases with both MPN and LPD would be expected. However, it has been shown that the presence of an MPN increases the probability of an LPD in the same individual. In a study by Vannucchi et al., which included 820 *BCR-/ABL1*-negative MPN patients in a period from 1980 to 2008, 11 concomitant LPD cases were reported, with a 3.44-fold increase in the risk of developing LPD in the MPN group compared with the general population [4]. In addition, Rumi et al. reported 22 concomitant LPD cases in a cohort of 1915 BCR-/ABL1-negative MPN patients followed up from 1970 to 2009, with a 2.79-fold increased risk, respectively [5].

Despite these observations, the coexistence of MPN and LPD remains rather rare; in fact, scarce case reports and limited cases series have been reported [6–11]. A recent systematic review of single patient clinical data by Marchetti et al. has identified 214 individuals harboring both diseases [12], while a Danish registry reported 97 new LPD cases in patients previously diagnosed with MPN [13].

Herein, we present our institution's experience with patients harboring both MPN and LPD, focusing on the peculiarities of their management. Furthermore, we suggest pathophysiological mechanisms that might explain the aforementioned coexistence.

## 2. Materials and Methods

A thorough search of the records of the Hematology Division of the Second Department of Internal Medicine and Research Institute at Attikon University General Hospital of Athens, Greece, was performed. The search included the records of all patients referred to our institution in the time period from 2003 to 2018. Clinical notes were used to identify patients harboring both diseases. Patient demographics, medical history, initial disorder at presentation, treatment for both diseases, and response to treatment were obtained from patient records. An informed consent was obtained from every subject.

## 3. Results

The search yielded nine cases of coexistent MPN and LPD out of a total of 269 patients diagnosed with *BCR-/ABL1*-negative MPN (244) or an MDS/MPN (myelodysplastic syndrome/myeloproliferative neoplasms (25)) and 1062 patients diagnosed with an LPD. Patient characteristics are depicted in Table 1.

Six out of the nine patients presented with a *BCR-/ABL1*-negative MPN. More specifically, one patient was diagnosed with PV, one patient with post-ET MF, two patients with primary myelofibrosis (PMF), and the remaining two with pre-fibrotic PMF; all of them were positive for the mutation *JAK2 V617F*. Of these patients, four required treatment with a cytoreductive agent, namely hydroxycarbamide (two of whom also required occasional bloodlettings), whereas one patient (Patient 8) received a JAK2 inhibitor (ruxolitinib) for 7 months. All MPN-treated patients had good control of their symptoms and blood counts.

Three out of the nine patients (patients 3, 6, and 7) were initially diagnosed as MDS-RS-T and were later found to fulfill criteria for MDS/MPN-RS-T according to the 2016 WHO revision [1]. None of them harbored the *JAK2 V617F *mutation; calreticulin (*CALR*) and *MPL W515* mutation studies were negative in Patient 6 but were not performed for patients 3 and 7 due to reimbursement issues. All of them were supported with an erythropoietin analogue and red blood cell transfusions.

Regarding the specific LPD phenotype, variability was noted as three patients were diagnosed with B-CLL and one patient with monoclonal B-cell lymphocytosis (MBL), three patients with lymphoma (two marginal zone lymphomas (MZL) and a plasmablastic lymphoma), and two patients with plasmacytic neoplasms (one with multiple myeloma and one with light and heavy chain deposition disease). All but two of the patients required treatment and were managed appropriately. The exact treatment approach for each patient is depicted in Table 1. Response was defined using the Cheson criteria [14] for patients 4 and 6. These criteria could not be applied for Patient 8 because even though lymphocytosis subsided with treatment, splenomegaly remained due to the underlying MPN. The International Workshop on Chronic Lymphocytic Leukemia (iwCLL) criteria [15] were used for patients 2 and 7. For Patient 9, the response was evaluated based on clinical criteria as discussed below.

All but one patient had significant comorbidities (Table 1). In the two patients with treatment intolerance, exacerbation of anemia was the main cause for stopping treatment. Both patients were old and frail. Patient 7 had been supported with RBC transfusions and erythropoiesis-stimulating agents for her MDS/MPN-RS-T. She only received 2 cycles of obinutuzumab/chlorambucil due to a dramatic increase in her transfusion needs. Her lymphocytosis has been in regression ever since. Patient 5 initially received hydroxycarbamide to control her thrombocytosis, but after 2 years, she developed anemia attributed to myeloma. A trial of lenalidomide was performed, which resulted in transfusion dependency, so treatment was stopped prematurely after a few days. On the other hand, the underlying MDS/MPN-RS-T did not seem to adversely affect treatment outcome of Patient 6, a fitter patient, who received 6 cycles of bortezomib/CHOP, resulting in an astonishing relapse-free survival, as previously reported [16].

It should be noted that the diagnosis of the MPN preceded that of LPD in all patients with the exception of Patient 4. Regarding this patient, a splenic MZL was diagnosed in another institution based on spleen pathology, and at this time point, bone marrow biopsy was reported to be normal and no further information was available in his records. Ten years later, the patient was referred to our institution for a massive abdominal lymphoid block which proved to be a relapse of the original low-grade LPD and for which he was treated with 8 cycles of rituximab. Myelofibrosis and *JAK2 V627* mutation were discovered during restaging of the patient. A few months later, a steady hemoglobin increase was observed and the patient was treated with hydroxycarbamide and phlebotomies. Whether this was a manifestation of his MPN is not known, as the patient (a heavy smoker) was also diagnosed with lung cancer shortly thereafter.

Patient 8 had suffered from ankylosing spondylitis the past 20 years and had received tumor necrosis factors (anti-TNF) to control her symptoms. She also received ruxolitinib for 7 months due to symptomatic splenomegaly. Baseline clonal immunoglobulin gene rearrangements and flow cytometry were not available for this patient before the initiation of ruxolitinib. She gradually developed anemia and clonal B-cell lymphocytosis. After 6 cycles of RCOP, both these findings have subsided. Ruxolitinib was not reintroduced for fear of LPD reoccurrence, as discussed below. Patient 2 received 6 cycles of obinutuzumab/chlorambucil and is currently on CR for his LPD.

Of special consideration is also Patient 9. This patient, having a 4-year history of JAK2-positive post-ET MF, developed persistent diarrheas and acute renal failure. Her serum protein electrophoresis and immunofixation were normal, and her bone marrow exhibited no plasmacytic infiltration. However, renal biopsy showed deposition of monoclonal immunoglobulin (IgAk), establishing the diagnosis of light chain and heavy chain deposition disease (LHCDD), also known as Randall disease, an entity recently recognized in the latest WHO classification [1]. Both diarrhea and acute renal failure subsided after six courses of bortezomib, cyclophosphamide, and dexamethasone. A repeat renal biopsy was not performed.

## 4. Discussion

We have presented a case series of patients with diverse MPN with or without myelodysplastic features coexisting with various LPD. To our knowledge, it is the first time that a patient with MPN and concomitant LHCDD has been reported. In our study, 3 MDS/MPN-RS-T patients with concomitant LPD are reported, explaining in part the relatively high prevalence of MPN/LPD in our population. Interestingly, there is a paucity of publications regarding the concomitance of MDS/MPN-RS-T with LPD, which suggests that MDS/MPN-RS-T might have been overlooked in previous studies.

Various points should be stressed regarding diagnosis and treatment of patients harboring both a MPN and a LPD. Regarding diagnosis, the presence of the *JAK2 V617F* mutation accompanied by flow cytometry-established B-cell clonality raises suspicion of coexistence of MPN and LPD. However, in *JAK2 V617F*-negative cases, a strict adherence to the WHO criteria for MPN, based on clinicopathological and molecular findings, is required.

The choice of treatment of one disease over the other is challenging. From a clinical point of view, it is often difficult to attribute features such as anemia, splenomegaly, or catabolic symptoms solely to one of the two entities. In such cases, the selection of treatment is guided by the assumed most symptomatic disease and further justified or not by the treatment outcome. In addition, and most importantly, treatment may be complicated by increased intolerance as reported for some of our patients. It has been shown that treatment of LPD may be poorly tolerated in patients with an underlying MPN. This is particularly true for cases with PMF or MDS/MPN overlap syndrome, who often present with anemia. In these cases, treatment of LPD could lead to an exacerbation of anemia due to decreased bone marrow reserve. Poor information is provided in the literature about treatment options and outcome of similar cases with coexistence of myeloid and lymphoid neoplasms. With the increasing awareness of the coexistence of the two entities, there is a growing need for optimizing treatment in these patients.

Several postulated mechanisms underlying the increased concomitance of MPN and LPD have been suggested. Notably, coexistence of a B-cell clone has been observed in MPN patients. Indeed, Pajor et al. [17] have shown that 5% of MPN patients harbor a clonal B-cell population as demonstrated with clonal immunoglobulin gene rearrangements. In addition, a coexisting B-cell clone was detected in 16.3% of myelofibrosis patients in a different study, in line with previous findings. Interestingly, when treated with JAK inhibitors, this subgroup of patients was at an increased risk of developing B-cell neoplasms [18]. In our cases series, a patient treated with JAK2 inhibitor, indeed, developed MZL; however, it should be noted that this patient's exposure to anti-TNF (tumor necrosis factor) agents for her ankylosing spondylitis could have also contributed to her lymphoma [19]. It should also be noted that whether JAK2 inhibitors are associated with an increased risk of lymphomagenesis remains equivocal, as the observational studies by Pemmaraju et al. [20] and Rumi et al. [21] did not show an increased risk of lymphoma in patients treated with these agents compared to those alternatively treated.

There is growing evidence that abnormalities in certain genes can result either in myeloid or in lymphoid malignancies. This is particularly true for the *BCR/ABL1* fusion gene and the *PDGFRA*, *PDGFRB*, and *FGFR1* genes [22] as well as for *TET2* mutations, observed in MDS, CMML, and in B and mainly T LPDs [23], for *SF3B1* mutations, associated with both the MDS-RS phenotype and B-CLL [24, 25].

Whether the two concomitant diseases originate in the same or in two different clones is difficult to postulate in the everyday clinical setting. In cases where both myeloid and lymphoid cells harbor the JAK2 mutation, a common *JAK2 V617F*-mutated progenitor probably exists [9]. On the other hand, when the mutation is not detected in the B cells, two separate clones are present at the same time [7, 8]. A possible explanation could be that a milieu of genomic instability exists, leading to the acquisition of a *JAK2 V617F* mutation in one clone and the development of a separate B-cell clone in the same patient [26].

The inflammatory bone marrow microenvironment could be the cause of such instability. In support of this notion, it has been shown that in PMF, clonal cells produce inflammatory cytokines, which in turn promote remodeling of the microenvironment in the abnormal niche [27]. Furthermore, in vivo experiments have demonstrated that tissues characterized by chronic inflammation promote specific B-cell tumorigenesis by providing an environment where neoplastic B cells escape normal regulatory mechanisms [28]. These two observations could explain two main findings of our study: the high prevalence of PMF in keeping with that observed by Porpaczy et al. [18] as well as the high rate of precedence of the MPN over the LPD.

There are several limitations in this study. Importantly, no extrapolation of the incidence of concomitant MPN and LPD in the entire Greek population can be made as this study includes a population from a single institution. Moreover, because of the retrospective nature of this study, some information regarding patient history and initial presentation is missing. Finally, a thorough molecular testing for these patients could not be done due to practical difficulties; certainly, the results of such testing would have shed light on the clonal association of the two neoplasms in these patients.

In conclusion, we have presented a relatively large number of patients with MPN and a coexistent LPD. Various difficulties pertain to the diagnosis and management of these patients. More research is needed to elucidate the underlying mechanisms predisposing for both MPN and LPD coexistence.

## Figures and Tables

**Table 1:** Patient characteristics and response to treatment.

Case Number	1	2	3	4	5	6	7	8	9
DOB	1942	1947	1930	1956	1930	1942	1924	1959	1955
Gender	Male	Male	Male	Male	Female	Male	Female	Female	Female
Comorbidities	CAD, AH	AH, BPH	CAD, peptic ulcer	AH, NSCLC, abdominal aneurysm	Dementia, thyroid goiter	AH, thoracic aneurysm	Hypothyroidism, glaucoma	Ankylosing spondylitis	Nonsignificant
MPN	Pre-fibrotic PMF	PV	MDS/MPN-RS-T	PMF	Pre-fibrotic PMF	MDS/MPN-RS-T	MDS/MPN-RS-T	PMF	Post-ET MF
*JAK2 V617F*	Positive	Positive	Negative	Positive	Positive	Negative	Negative	Positive	Positive
MPN treatment	Observation	Hydroxycarba mide, bloodlettings	Epoetin alpha, RBC transfusions	Hydroxycarbamide, bloodlettings	Hydroxycarbamide	Epoetin alfa, RBC transfusions	Epoetin alpha, RBC transfusions	Ruxolitinib	Hydroxycarbamide
Age at MPN	74	67	84	60	85	74	85	53	59
LPD	MBL	B-CLL	B-CLL	MZL	MM	PBL	B CLL	Splenic MZL	LHCDD
Age at LPD	74	70	86	50	86	76	93	57	63
LPD treatment	Observation	Obinutuzumab/Chlorambucil	Observation	Splenectomy, Rituximab	Lenalidomide	Bortezomib-CHOP	Obinutuzumab/Chlorambucil	RCOP	VCD
LPD treatment outcome	NA	CR	NA	PR	Intolerance	CR	Intolerance	CR	CR

DOB: Date of birth, MBL: Monoclonal B-cell lymphocytosis, MF: Myelofibrosis, B-CLL: B chronic lymphocytic leukemia, MM: Multiple myeloma, LHCDD: Light and heavy chain deposistion disease RBC: Red blood cell, MDS/MPN RS-T: Myelodysplastic syndromes/myeloproliferative neoplasms with ring sideroblasts and thrombocytosis, CAD: Coronary artery disease, AH: Arterial hypertension, BPH: Benign prostate hyperplasia, NSCLC: NonSmall cell lung carcinoma, CR: Complete response, PR: partial response, MZL: Marginal zone lymphoma, PBL: Plasmablastic lymphoma, VCD: Bortezomib, cyclophosphamide, dexamethasone, RCOP: Rituximab cyclophosphamide vincristine prednisolone CHOP: Cyclophosphamide, doxorubicin, vincristine, prednisolone, NA: Nonapplicable.

## Data Availability

The data used to support the findings of this study are available from the corresponding author upon request.

## References

[1] Swerdlow S. H., Campo E., Harris N. L. (2017). *WHO Classification of Tumours of Haemaotopoietic and Lymphoid Tissues*.

[2] Moulard O., Mehta J., Fryzek J., Olivares R., Iqbal U., Mesa R. A. (2014). Epidemiology of myelofibrosis, essential thrombocythemia, and polycythemia vera in the European Union. *European Journal of Haematology*.

[3] Redaelli A., Laskin B. L., Stephens J. M., Botteman M. F., Pashos C. L. (2004). The clinical and epidemiological burden of chronic lymphocytic leukaemia. *European Journal of Cancer Care*.

[4] Vannucchi A. M., Masala G., Antonioli E. (2009). Increased risk of lymphoid neoplasms in patients with Philadelphia chromosome-negative myeloproliferative neoplasms. *Cancer Epidemiology Biomarkers and Prevention*.

[5] Rumi E., Passamonti F., Elena C. (2011). Increased risk of lymphoid neoplasm in patients with myeloproliferative neoplasm: a study of 1,915 patients. *Haematologica*.

[6] Chintapatla R., Battini R., Wiernik P. H. (2012). Chronic lymphocytic leukemia with essential thrombocythemia: asbestos exposure association?. *Clinical Advances in Hematology & Oncology*.

[7] Henry L., Carillo S., Jourdan E., Arnaud A., Brun S., Lavabre-Bertrand T. (2007). Association of essential thrombocythemia and chronic lymphocytic leukemia: absence of the V617F JAK2 mutation in the lymphoid compartment. *American Journal of Hematology*.

[8] Hussein K., Brakensiek K., Ballmaier M. (2006). B-CLL developing in a patient with PV is not affected by V617F mutation of the Janus kinase 2. *European Journal of Haematology*.

[9] Kodali S., Chen C., Rathnasabapathy C., Wang J. C. (2009). JAK2 mutation in a patient with CLL with coexistent myeloproliferative neoplasm (MPN). *Leukemia Research*.

[10] Mousinho F., Santos P. S. E., Azevedo A. P. (2018). Concomitant myeloproliferative and lymphoproliferative neoplasms, distinct progenitors: a case report and review of the literature. *Molecular and Clinical Oncology*.

[11] Trifa A. P., Cucuianu A., Popp R. A. (2014). Concomitant myeloproliferative and lymphoid neoplasms in two patients positive for JAK2 V617F mutation. Case report and literature review. *Indian Journal of Hematology and Blood Transfusion*.

[12] Marchetti M., Carobbio A., Capitoni E., Barbui T. (2018). Lymphoproliferative disorders in patients with chronic myeloproliferative neoplasms: a systematic review. *American Journal of Hematology*.

[13] Holst J. M., Plesner T. L., Pedersen M. B. (2017). Lympho- and myeloproliferative malignancies occurring in the same host: description of a nationwide discovery cohort. *Blood*.

[14] Cheson B. D., Fisher R. I., Barrington S. F. (2014). Recommendations for initial evaluation, staging, and response assessment of hodgkin and non-hodgkin lymphoma: the lugano classification. *Journal of Clinical Oncology*.

[15] Hallek M., Cheson B. D., Catovsky D. (2018). iwCLL guidelines for diagnosis, indications for treatment, response assessment, and supportive management of CLL. *Blood*.

[16] Bouchla A., Papageorgiou S. G., Tsakiraki Z. (2018). Plasmablastic lymphoma in an immunocompetent patient with MDS/MPN with ring sideroblasts and thrombocytosis—a case report. *Case Reports in Hematology*.

[17] Pajor L., Lacza Á., Kereskai L. (2004). Increased incidence of monoclonal B-cell infiltrate in chronic myeloproliferative disorders. *Modern Pathology*.

[18] Porpaczy E., Tripolt S., Hoelbl-Kovacic A. (2018). Aggressive B-cell lymphomas in patients with myelofibrosis receiving JAK1/2 inhibitor therapy. *Blood*.

[19] Mariette X., Tubach F., Bagheri H. (2010). Lymphoma in patients treated with anti-TNF: results of the 3-year prospective French RATIO registry. *Annals of the Rheumatic Diseases*.

[20] Pemmaraju N., Kantarjian H., Nastoupil L. (2019). Characteristics of patients with myeloproliferative neoplasms with lymphoma, with or without JAK inhibitor therapy. *Blood*.

[21] Rumi E., Zibellini S., Boveri E. (2019). Ruxolitinib treatment and risk of B-cell lymphomas in myeloproliferative neoplasms. *American Journal of Hematology*.

[22] Bain B. J. (2010). Myeloid and lymphoid neoplasms with eosinophilia and abnormalities of PDGFRA, PDGFRB or FGFR1. *Haematologica*.

[23] Lemonnier F., Couronne L., Parrens M. (2012). Recurrent TET2 mutations in peripheral T-cell lymphomas correlate with TFH-like features and adverse clinical parameters. *Blood*.

[24] Yoshimi A., Abdel-Wahab O. (2017). Splicing factor mutations in MDS RARS and MDS/MPN-RS-T. *International Journal of Hematology*.

[25] Wan Y., Wu C. J. (2013). SF3B1 mutations in chronic lymphocytic leukemia. *Blood*.

[26] Tabaczewski P., Nadesan S., Lim S. H. (2009). Zap-70 positive chronic lymphocytic leukemia co-existing with Jak 2 V671F positive essential thrombocythemia: a common defective stem cell?. *Leukemia Research*.

[27] Le Bousse-Kerdilès M.-C. (2012). Primary myelofibrosis and the “bad seeds in bad soil” concept. *Fibrogenesis & Tissue Repair*.

[28] Cain D., Kondo M., Chen H., Kelsoe G. (2009). Effects of acute and chronic inflammation on B-cell development and differentiation. *Journal of Investigative Dermatology*.

